# The Frontal Area with Higher Frequency Response Is the Principal Feature of Laser-Evoked Potentials in Rats with Chronic Inflammatory Pain: A Parallel Factor Analysis Study

**DOI:** 10.3389/fneur.2017.00155

**Published:** 2017-05-08

**Authors:** Jing Wang, Juan Wang, You Wan, Xiaoli Li

**Affiliations:** ^1^Department of Neurobiology, School of Basic Medical Sciences, Beijing Institute for Brain Disorders, Capital Medical University, Beijing, China; ^2^Institute of Electrical Engineering, Yanshan University, Qinhuangdao, China; ^3^Key Laboratory for Neuroscience, Ministry of Education/National Health and Family Planning Commission, Beijing, China; ^4^State Key Laboratory of Cognitive Neuroscience and Learning, IDG/McGovern Institute for Brain Research, Beijing Normal University, Beijing, China

**Keywords:** chronic inflammatory pain, event-related potentials, parallel factor analysis, wavelet transform, rat

## Abstract

Chronic pain is a pathological developing course of pain. In clinic, an objective indicator is needed for diagnosing and better controlling chronic pain. The abnormal neural responses in chronic pain are reflected by multiple event-related potentials (ERPs) in time, frequency, and location domain, respectively. However, multiple changes in ERPs are not applicable in clinic. So, the principal feature covered the most informative changes extracted from these three domains of ERP during the development of chronic pain is needed. In the present study, a parallel factor analysis method was employed to extract time–frequency–channel features of laser-evoked potential (LEP) simultaneously from rats with chronic inflammatory pain. Results showed that the main feature of LEP in channel domain locates in the frontal brain region in rats with chronic inflammatory pain while in the parietal brain region in control rats. In the frequency domain, the main frequency of LEP was significantly higher in chronic inflammatory pain rats than that in control rats. These findings indicate that the frontal region with higher frequency response to nociceptive information is the principal feature in the chronic pain state. Our study provided not only a principal feature of LEP but also a promising strategy for chronic pain, which is potential for clinic application.

## Introduction

Chronic pain is a pathological pain state characterized by pain persistence (1). It is believed that chronic pain is not a simple condition of persistent pain perception, but a course of pain chronification that involves sensation, emotion, and cognition (2, 3). Clinically, diagnosis of chronic pain mainly relies on subjective pain report lasting for several months; however, dependence of subjective pain report results in unsatisfied treatment of pain in clinic because of missing the optimal treatment window. Detection of chronic pain with objective measurement at early stage, but not with traditional subjective report is required (4). Thus, it is of importance to explore a measurement or an indicator that is applicable in clinic.

Event-related potentials (ERPs) are a measurement that reflects neuronal processes by frequency, time course, and topography changes (5). ERPs have been applied to study the abnormal neural response in chronic pain conditions (5–7). Most studies employ painful laser stimuli to evoke cortical neural responses that are named laser-evoked potential (LEP). It is widely used in chronic pain clinically (8). Previous researches indicated that LEP in the time, location, and frequency domain was altered in chronic pain. For example, one study found that oscillatory activities in the theta frequency band were enhanced in chronic pain patients in parietal areas (9), indicating changes in the frequency and location domain. In another study, the N170 component (a negative potential appeared at 170 ms after stimulation) of LEP was enhanced in the fronto-central region (10), showing alternation in the time domain at another location domain. In addition, chronic pain patients showed both changes for the N1 component (a negative potential appeared at 100 ms after stimulation) in the temporal region and the N2/P2 component in the vertex region (11). These results demonstrate that multiple time and frequency responses recorded at a number of regions alter in chronic pain condition. Although these multiple changes are sufficient for understanding the mechanism of chronic pain, it is still unclear which are the principal characteristics covered the most informative changes related with chronic pain, and it is not suitable for clinic application. Therefore, a principal feature extracted from abundant information is required for clinic usage.

Effective reduction methods such as principal component analysis (PCA) are traditionally applied for ERPs to explore the principal components and to characterize ERPs (12, 13). However, the PCA method only describes the time and frequency characteristics within single recording area. It cannot extract components for multichannel ERPs (14). The parallel factor analysis (PARAFAC) is a method that could extract features in the time–frequency–channel domain simultaneously from original multichannel EEG data (15). It takes into account the frequency of oscillations in certain time periods among all the recording channels (16) and has been successfully applied to detect abnormal oscillatory activity in epilepsy and Alzheimer’s disease (17).

Therefore, in the present study, in order to explore the principal feature of LEP during the development of chronic inflammatory pain in three domains, we recorded LEP obtained from the electrocorticogram (ECoG) of rats with chronic pain model and applied the PARAFAC method to decompose multichannel LEP data. Components of each rat are the main characteristics of the time–frequency–channel information for brain oscillatory activities.

## Animals and Methods

### Animals

Thirteen male Sprague-Dawley adult rats (weight 300–350 g) were used. These animals were provided by the Department of Experimental Animal Sciences, Peking University Health Science Center. The animals were housed individually in cages at room temperature of 22 ± 1°C and kept on a 12 h light and dark cycle. Food and water were available *ad libitum*. The rats were allowed to habituate to the environment and handled by the experimenter daily for 1 week before surgery.

### Surgery and Electrodes Implantation

**Figure 1:**
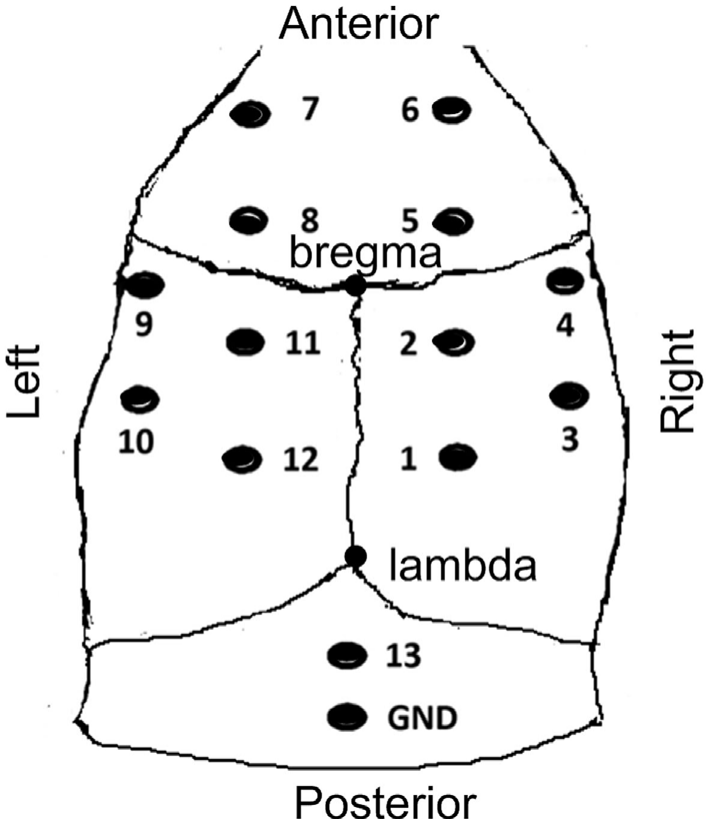
**The sketch map of electrodes locations**. Anterior to the bregma (A+); posterior to the bregma (A−); left lateral to the bregma (L+); right lateral to the bregma (L−); electrodes 4 and 9 (A 0.0 mm, L ±4.5 mm); electrodes 3 and 10 (A −3.0 mm, L ±4.5 mm); electrodes 6 and 7 (A +4.5 mm, L ±1.5 mm); electrodes 8, 5, 11, 2 (A ±1.5 mm, L ±4.5 mm); electrodes 1 and 12 (A −4.5 mm, L ±1.5 mm); electrode 13 operated as the reference electrode. The reference electrode and ground electrode were positioned 2 and 4 mm caudal to lambda, respectively.

Rats were anesthetized with sodium pentobarbital (50 mg/kg, *i.p*.). Fourteen stainless steel screws (tip diameter 1 mm, impedance of 300–350 Ω, Kanpu Medical Ltd., China) equipped with a socket were implanted as epidural electrodes on the skull. The locations of the electrodes were determined by the method proposed by Shaw (18), as shown in Figure 1. The electrodes were fixed to the skull with dental cement. Penicillin (60,000 U, *i.m*.) was administrated in the following 3 days after operation to prevent possible infection. The EEG recording was started 7 days after electrodes implantation operation.

### Chronic Inflammatory Pain Model of Rats: Monoarthritis Procedure

A chronic inflammatory pain model of monoarthritis in rats was established according to a previously described method by Butler (19). The procedure was briefly as follows: the complete Freund’s adjuvant (CFA) component includes *Mycobacterium butyricum* (60 mg), paraffin oil (6 ml), NaCl 0.9% (4 ml), and Tween 80 (1 ml). After being mixed and autoclaved, the mixture (0.05 ml) was injected into the left tibiotarsal joint cavity to induce the monoarthritis pain model (CFA group). Six rats in the control group were injected with an equal volume of vehicle (0.9% NaCl) to the left tibiotarsal joint cavity [normal saline (NS) group]. After injection, rats in the CFA group developed thermal hyperalgesia, which is confirmed by the rat’s hind paw withdrawal response to a lower laser intensity compared with that before injection.

### Laser Stimulation

On the day prior to intra-articular injection (D0) and 1, 7, 14, and 28 days after intra-articular injection (D1, D7, D14, and D28), rats received thermal nociceptive stimulation with a laser beam (wavelength 10.6 µm, beam diameter 2.5 mm, pulse width 20 ms), which was delivered by a CO_2_-laser stimulator (DIMEI-300, Changchun Optics Medical Apparatus Co., Ltd., China). Laser stimuli were applied to the plantar of hind paw when rats were awake and quiet. The appropriate intensity of the laser beam for each individual rat was determined by using an ascending series of laser beam intensities with a 1 W increment. The intensity that generated four to five hind paw withdrawal responses out of six stimuli was selected as the intensity of stimulation. Each rat received 15 stimuli that could induce hind paw withdrawal responses. Each stimulus was targeted at slightly different positions. The interstimulation interval varies from 40 to 150 s.

### Recordings of Laser-Evoked ECoG

The EEG/ERP system (CogniTrace ERP, ANT Inc., The Netherlands) was used for EEG data collection. Twelve recording electrodes and one reference electrode were connected to the digital preamplifier, and the ground electrode was connected the GND connector of the amplifier. All signals were referenced to the electrode that was located 2 mm caudal to lambda (the #13 electrode in Figure 1). The sampling frequency of EEG recording was 1,024 Hz. Rat behaviors were videotaped while the ECoG was recording.

### Preprocessing

The preparation and preprocessing of data were carried out as follows. The duration of each epoch was set as 1,500 ms (500 ms before and 1,000 ms after the laser stimulation onset). Large baseline drift was checked and removed for all trials and channels. Then, the epoch signals were re-referenced to an average of all channel recordings. Finally, the laser-evoked ECoG data were preprocessed with a band-pass filter of 1–70 Hz (eegfilter.m at EEGLAB software: http://sccn.ucsd.edu/eeglab/), and the ERP data were generated by averaging.

### Wavelet Transforms and PARAFAC

Wavelet transformation and PARAFAC were performed as described by Wang (16). Briefly, first, wavelet transforms were used to transform ERP into time-frequency energy for each channel, obtaining a time × frequency matrix. Next, matrices from 12 channels were put together to form a dataset containing time, frequency, and channel information. Then, PARAFAC method was applied to this dataset to obtain its main component. PARAFAC projects the dataset in the time domain, frequency domain, and channel domain and extract several orthogonal components to maximally represent the original dataset. The number of components is controlled by core consistency method (17, 20). After PARAFAC, components consisting of three coefficients matrices, i.e., matrix of time, frequency, and channel was obtained, reflecting the main characteristics in the time, frequency, and channel domain, respectively.

Then, the time points with biggest coefficients and the frequencies with biggest coefficients were selected. For further channel domain analysis, the coefficients of every channel were plotted.

### Statistical Analysis

Two-way analysis of variance was used to compare the frequency and time difference between the two groups.

## Results

### PARAFAC Analysis of LEP

**Figure 2:**
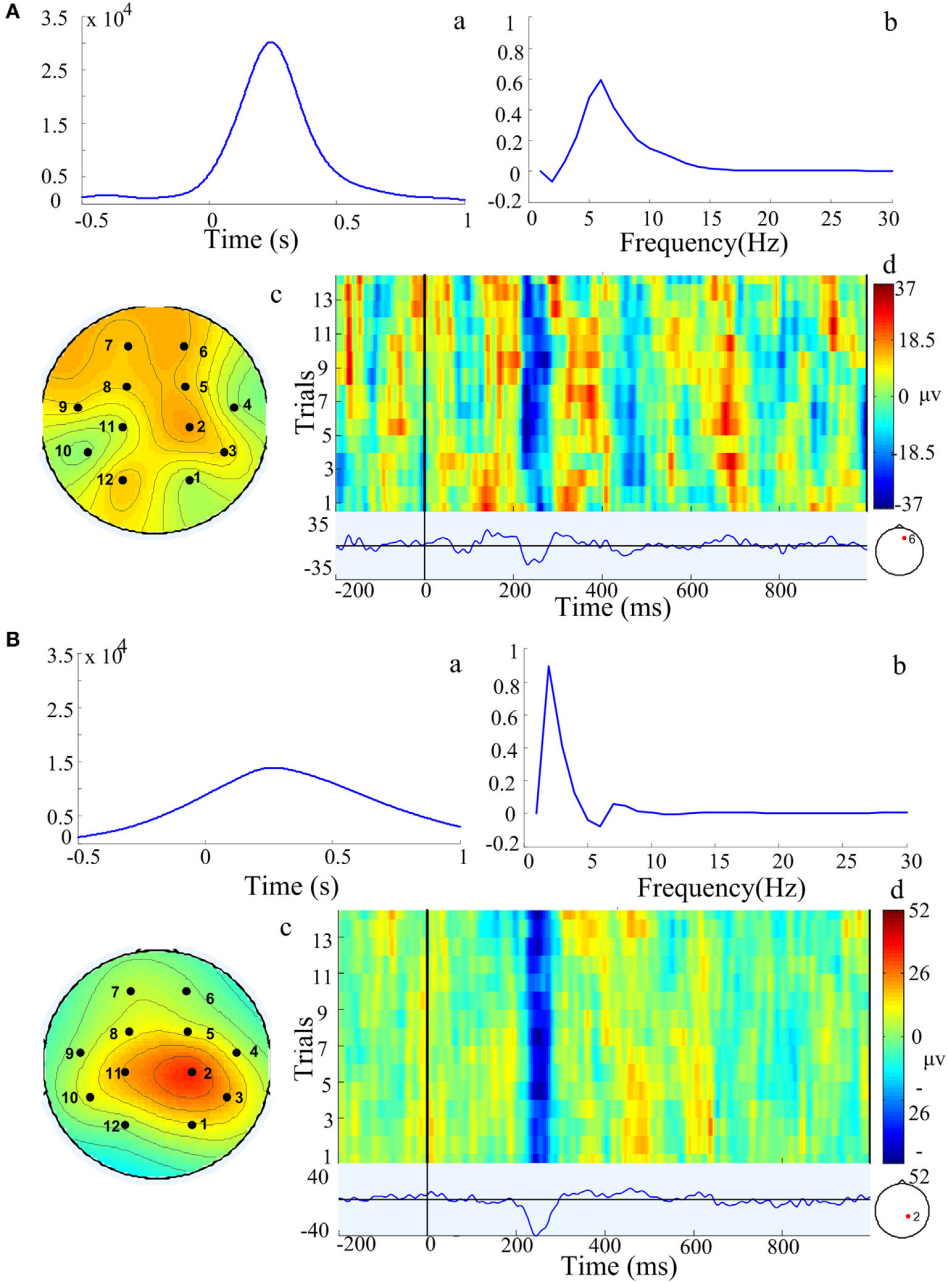
**Components extracted by the parallel factor analysis (PARAFAC) method model from one representative example in a chronic arthritic rat**. Two components **(A,B)** were extracted by PARAFAC. In each component, it contained the characteristic in time domain (a), frequency domain (b), and location domain (c) of three-dimensional information; (d) was the corresponding laser-evoked potential and stimulation trials from a single channel marked with red point in a chronic arthritic rat. Based on the peak of these coefficients, component **(A)** is over frontal region with a frequency of 6 Hz at approximately 240 ms after the stimulus onset; component **(B)** is over parietal region with a frequency of 2 Hz at 250 ms after stimulation onset.

In order to test the accuracy of PARAFAC method, the feature extracted by PARAFAC was compared with original LEP. With PARAFAC analysis, one or more components that represent main characteristics of EEG are obtained for each data. Within each component, it contains three-way information in the time–frequency–channel domain. A representative example with the PARAFAC analysis is shown in Figure 2. These data have two components, which are shown in Figures 2A,B, respectively. In each component, three-dimensional information at the time–frequency–channel domain was obtained. The information in the first component is shown in Figure 2A, a–c. Based on the peak of coefficients, the main characteristic of this multichannel EEG signal was located in the prefrontal region with a frequency of around 6 Hz at approximately 240 ms after the stimulus onset. The corresponding LEP obtained by across-trial averaging in EEGLAB from the channel 6 is plotted in Figure 2A, d. It was obvious that the time feature extracted by the PARAFAC method (~240 ms, seeing Figure 2A, a) matched well with the LEP latency in channel 6 in the prefrontal region (~250 ms, seeing Figure 2A, d). For the second component, the decomposition is shown in Figure 2B, a–c. The main characteristic of this multichannel EEG signal was located in the parietal region with frequency around 2 Hz at approximately 250 ms after the stimulus onset. From the LEP waveform in channel 2 in parietal region, the latency of the LEP was at around 250 ms in the parietal region (Figure 2B, d).

### The Feature of LEP during the Development of Chronic Pain

Table 1 showed that pain threshold, measured by laser intensity that induces rat’s hind paw withdrawal response, was lower in rats with CFA than rats with NS [*F*_group(1,44)_ = 24.48, *P* < 0.001], indicating that rats with CFA injection developed thermal hyperalgesia. *Post hoc* analysis revealed lower threshold at all days in rats with CFA (*P* < 0.05 for D1, D7, D14, and D28).

**Table 1:** **Changes of pain threshold relative to baseline (D0)**.

Time					
Group	D0 (%)	D1 (%)	D7 (%)	D14 (%)	D28 (%)
CFA group*	100	−32.4 ± 12.1	−28.0 ± 15.7	−35.0 ± 19.0	−48.9 ± 15.2
NS group	100	−4.9 ± 12.0	−5.3 ± 7.6	−6.2 ± 6.5	−6.5 ± 5.8

*Data are expressed as mean ± SEM*.

*CFA, complete Freund’s adjuvant; NS, normal saline*.

*P < 0.05 for all comparisons 1, 7, 14, and 28 days after injection by post hoc analysis.

After the PARAFAC analysis, there were several components extracted by each rats. Each component consisted of three coefficients matrices, i.e., matrix of time, frequency, and channel, reflecting the main characteristics in the time, frequency, and channel domain, respectively. We further collected all the components in each group and then compared each of the three domains between two groups.

**Figure 3:**
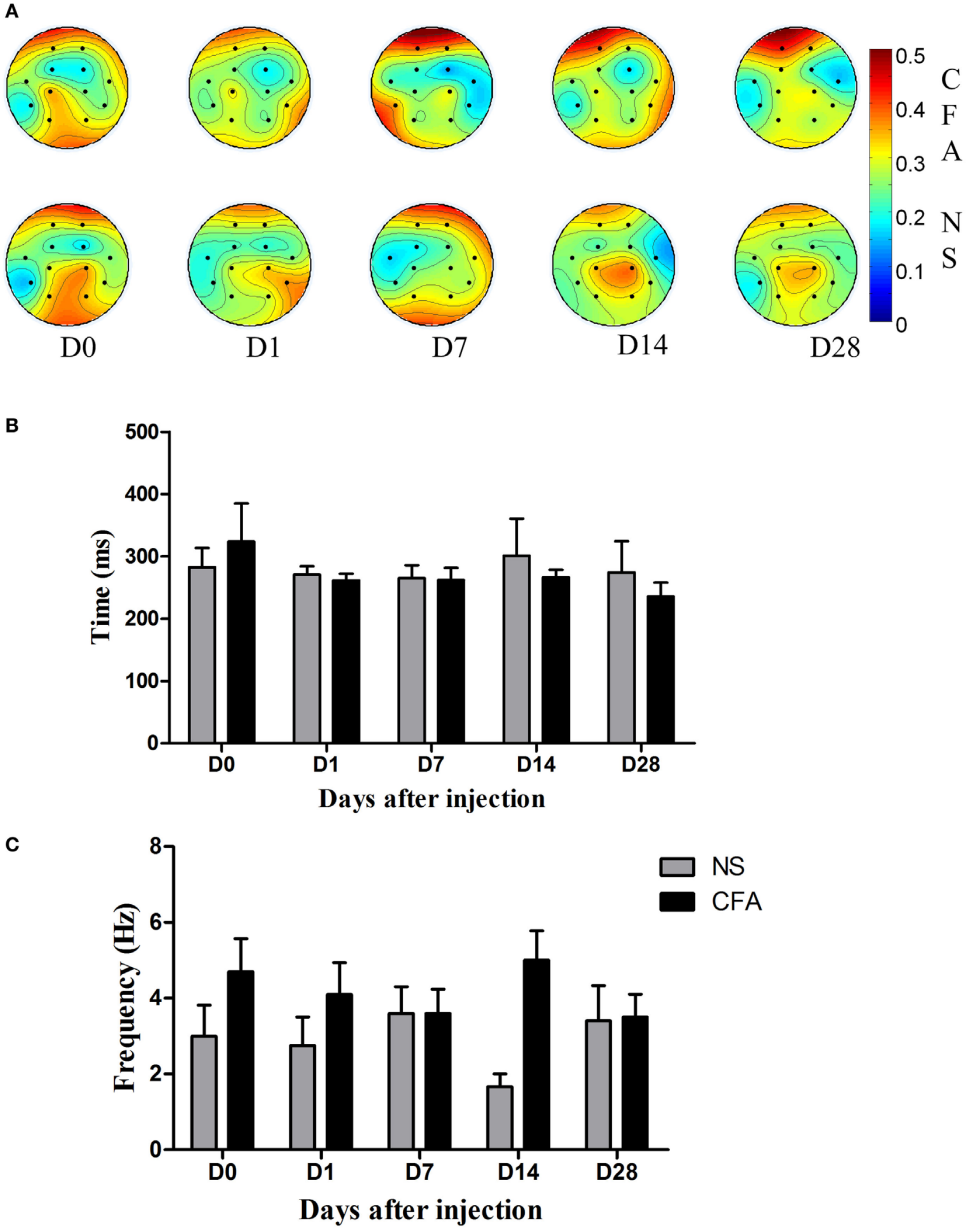
**The channel, frequency, and time characteristics in the complete Freund’s adjuvant (CFA) induced monoarthritis and normal saline (NS) control groups**. **(A)** Topography of channel coefficients. The upper picture was average value of coefficients for each channel in control rats at days 0, 1, 7, 14, and 28. The lower picture was the average value of coefficients for each channel in rats with chronic pain at days 0, 1, 7, 14, and 28. **(B)** The results of the time feature in comparison between the CFA pain and NS control groups at days 0, 1, 7, 14, and 28. There is no difference between groups at each time point in the time domain. **(C)** The results of the frequency feature in comparison between the CFA pain and NS control groups at days 0, 1, 7, 14, and 28. There is main effect of group [*F*_group(1,69)_ = 5.3, *P* = 0.024], indicating the frequency response to the nociceptive stimulation was higher in the CFA group than that in the NS group.

To find out the difference in the channel domain between the CFA and NS groups, coefficients of each channel from both the NS and CFA groups at different days before and after pain induction were plotted (Figure 3A). In the CFA group, we found the activity over the prefrontal region increased along the development of chronic pain. The coefficients of the channel 7 were bigger at day 7 (*t* = 2.21, *P* < 0.05) and day 28 (*t* = 2.69, *P* < 0.05) compared with the coefficients of channel 7 at day 0. Unlike the CFA group, no channels showed difference among all these days in the NS group. The topographic difference between the two groups indicates main feature of LEP locates in the frontal regions in the chronic pain.

The difference in the time domain and the difference in the frequency domain between the two groups were plotted in Figures 3B,C, respectively. We found that there was no difference between groups at each time point in the time domain [*F*_group(1,69)_ = 0.63, *P* = 0.42]. The time information of both groups was around 250 ms.

In the frequency domain, there was a statistically significant higher frequency response in the CFA group than the NS groups [*F*_group(1,69)_ = 5.3, *P* = 0.024]. *Post hoc* analysis revealed no specific day showed difference between groups (*P* > 0.05).

## Discussion

### PARAFAC Method

Previous studies showed that the PARAFAC was a promising approach to process multiple channel EEG signals (21) and has been successfully used to characterize the structure of epileptic EEG data (17, 22, 23). In this study, the PARAFAC method was used to extract the information in the time–frequency–channel from the ERPs of rats with chronic pain. The feature extracted by PARAFAC was around 250 ms in the prefrontal and parietal regions. It is noticeable that the feature matches with the corresponding ERP results (Figure 2A, d and Figure 2B, d) and with previous LEP findings that the N2/P2 complex components peaks in the frontal region at approximately 250 ms after stimulus onset (24). These results provided further evidence as suggested by Wang (16) that the PARAFAC is an interesting method to detect the principal character of LEP.

### Nociception-Corrected LEP during the Development of Chronic Pain

Laser-evoked potential is dependent on intensity of nociceptive perception (25). Equivalent stimulus intensity leads to significantly different intensity of nociceptive perception between CFA rats and control due to the thermal hyperalgesia in chronic pain. In order to eliminate the influence of stimulus-induced perceptional intensity on the laser-evoked potentials, we corrected the laser intensity for both CFA and NS group based on their nociceptive behavioral response. Laser intensities that produced equivalent nociceptive perception for both CFA and NS rats were used. Thus, the difference of LEP in our study between chronic pain and control is more likely reflect the innate difference in the neural system instead of the difference due to the external stimulus-induced perception.

Based on the definition of chronic pain that pain lasts even after the original tissue damage has cured, the nature of chronic pain is the persistence of pain. Traditional researches that study changes at one time point of chronic pain are limited for providing the ongoing and developmental characteristic of chronic pain. In this study, we explored longitudinal study to find the dynamic changes of ERP features during the development of chronic pain.

### Location Feature of LEP during the Development of Chronic Pain

The frontal and parietal brain regions were found to be the principal feature response to the laser nociceptive stimulation at day 0 (Figure 3A). The parietal region is shown to process the sensory component of pain information (26, 27). Besides, the frontal area, the location of the anterior cingulate cortex and prefrontal cortex, which are mainly responsible for in the emotional and cognitive aspect of pain (28), is also involves in processing the laser nociceptive information. This result is in accordance with previous findings in healthy people, showing the sources of the LEP were located in the primary somatosensory cortex of parietal region (28, 29).

Results showed that the coefficients in the frontal region increased in rats with chronic inflammatory pain but did not change in the control rats. It indicates rats with chronic inflammatory pain process the nociceptive information predominantly in the frontal regions. It was in line with previous functional magnetic resonance imaging and electrophysiology studies indicating activity in the prefrontal region was increased in chronic pain condition (9, 30). The difference in channel domain between the control rats and the rats with chronic pain suggests the network for processing pain information changes as chronic pain develops—from the sensory dominant network centered at the parietal brain region to the affective dominant network centered at the frontal region. This dynamic change of network over time supports the idea that the affective aspect of pain gradually outweighs the sensory aspect of pain in the development of chronic pain (31).

The contribution of the frontal region seemed to become smaller over recording days in the NS control group though statistic results showed no difference. This phenomenon was probably due to pain habitation, i.e., reduction of pain and pain-related response by repetitive stimulation (6, 32). Rennefeld reported that pain rating of healthy people was reduced after several days of repeated painful stimulation, indicating subjects are habituation to pain (32). During our experiment, rats received repetitive painful stimuli and became familiar with the nociceptive stimulation and the experimental procedures over time, so the unpleasant component reflected in the frontal region should have decreased gradually due to pain habitation. Interestingly, we found rats with chronic pain did not exhibit reduction of the frontal region activation as control rats did (Figure 3A). It suggests lack of habituation to pain in chronic pain condition.

### Frequency Feature of LEP during the Development of Chronic Pain

In the frequency domain, the frequency response was found to be higher in rats with CFA than the control. It is consistent with previous finding that the frontal network predominantly oscillated at higher frequencies in chronic pain (33). It was reported that activity at both delta and theta frequency band (1–3 and 3–8 Hz) was associated with P300 component of ERP (34–36). From this perspective, these frequency responses are all belong to the P300 component. In view of the idea that higher frequency oscillations are incline to promote neural synchronization within focal areas and facilitate neural plasticity than lower frequency (37), the higher frequency in the frontal region in our results is probably related to neural sensitization of local brain region in the chronic pain condition. Results from *in vitro* studies demonstrating that synaptic plasticity and neural sensitization occur in frontal cortex under chronic pain conditions (38, 39) support this explanation. Although the frequency of ERP could provide extra information that overlaps in traditional time domain studies, the meaning of the frequency of ERP is unclear yet. Therefore, whether the difference between two groups in our results is physiological significance deserves further work. Anyway, our results provide a more detailed characterization of LEP in chronic pain.

Frequency response is usually studied as frequency bands, which could eliminate the individual variation. However, the edge of frequency band is artificial, and the trend of researches is to apply the dominant peak frequency rather than frequency band (40). In our study, we performed specific dominant frequency. Accordingly, it could be a reason for 1 Hz variations in the frequency domain shown at day 0 between two groups and at day 14 between other days.

### Limitations and Future Directions

Our exploratory research has several limitations. First, LEP from ECoG of rats with chronic pain could not be transformed to patients, though rats study allows longitudinal study and high ratio of signal to noise. Further experiment from patients and ongoing pain would be performed. Second, the limited spatial resolution of rat ECoG and PARAFAC method is not allowed to be focused on specific brain areas, which is hard to explain the underlying neurophysiology mechanism. Third, the casual and unique relationship between these features and chronic pain is hardly determined from the current study. Intervention research is needed in future. Fourth, small sample size is a weak point because of long-time recordings.

In conclusion, we applied the PARAFAC analysis in the multichannel LEPs during the development of chronic pain. It was found that the frontal region with higher frequency was the principal feature of neural response in the chronic pain condition. These features provide a potential neural network characteristic for chronic pain. Besides, our study provides a promising strategy of applying LEP combined with PARAFAC for assisting chronic pain diagnosis and treatment.

## Ethics Statement

All animal experiments were conducted with approval of the Animal Care and Use Committee of our university and were in accordance with the Guidelines of International Association for the Study of Pain.

## Author Contributions

JW (first author) carried out the ECoG recording and data analysis, participated in the study design, and drafted the manuscript. JW (second author) carried out the data analysis. YW participated in the study design and helped to draft the manuscript. XL conceived of the study, participated in its design, and helped to draft the manuscript. All the authors read and approved the final manuscript.

## Conflict of Interest Statement

The authors declare that the research was conducted in the absence of any commercial or financial relationships that could be construed as a potential conflict of interest.
